# Identify novel therapeutic targets for type II diabetes and periodontitis: insights from single-cell analysis and Mendelian randomization analysis

**DOI:** 10.3389/fendo.2024.1410537

**Published:** 2024-10-31

**Authors:** Mingrui Zou, Jichun Yang

**Affiliations:** ^1^ Department of Physiology and Pathophysiology, School of Basic Medical Sciences, State Key Laboratory of Vascular Homeostasis and Remodeling, Center for Non-coding RNA Medicine, Peking University Health Science Center, Beijing, China; ^2^ Peking University First School of Clinical Medicine, Peking University First Hospital, Beijing, China

**Keywords:** GWAS, Mendelian randomization, periodontitis, single-cell analysis, type II diabetes

## Abstract

**Background:**

Periodontitis is a common complication of type II diabetes (T2D). However, the existing research cannot fully elucidate the association between them, let alone identify therapeutic targets for precise treatment of diabetic periodontitis. Therefore, we employed integrated genetic approaches such as single-cell analysis, Mendelian randomization (MR) analysis and colocalization analysis to uncover novel therapeutic targets for T2D and periodontitis.

**Methods:**

This study integrated single-cell analysis, MR analysis, colocalization analysis, phenotype scanning, cell-cell communication analysis and metabolic pathway activity analysis to unveil novel therapeutic targets for periodontitis and T2D. We firstly identified core cell clusters of T2D and periodontitis, and important marker genes were selected. The causal associations between these genes and the two diseases were evaluated through MR analysis. Reverse MR analysis, colocalization analysis, additional validation and phenotype scanning further supported our findings. Finally, cell-cell communication analysis and metabolic pathway activity analysis were employed to preliminarily investigate the mechanisms of the observed causal associations.

**Results:**

Through analysis of scRNA-seq data, we identified classical monocytes and intermediate monocytes as core cell subclusters. Differential analysis identified 221 differentially expressed genes (DEGs). MR analysis identified 13 genes exhibiting causal associations with T2D, and 11 causal genes with periodontitis. Colocalization analysis, reverse MR analysis, additional validation and phenotype scanning further enhanced the robustness of our results. Finally, we identified NCF1 as the core therapeutic target for T2D (OR = 1.09, 95% CI: 1.03-1.14, *p* = 1.85 $\;\times 10^{-3}$ ) and LRRC25 for T2D (OR = 0.96, 95% CI: 0.93-0.99, *p* = 3.44 $\;\times 10^{-2}$ ) and periodontitis (OR = 0.92, 95% CI: 0.84-0.99, *p* = 4.45 $\;\times 10^{-2}$ ). At last, cell-cell communication analysis indicated significant differences in functions and metabolic pathway activity between monocytes expressing or not expressing the core causal genes, which preliminarily interpreted the observed causal associations.

**Conclusion:**

This study integrated single-cell analysis, MR analysis and colocalization analysis to identified novel therapeutic targets for T2D and periodontitis. 13 causal genes were identified for T2D, and 11 for periodontitis. Among them, NCF1 and LRRC25 were regarded as core therapeutic targets. Our findings bridge the gap in the understanding of the association between T2D and periodontitis, and pave the way for targeted therapy of the two diseases.

## 1 Introduction

Type II diabetes (T2D), a common chronic metabolic disease, is characterized by insulin resistance (IR) and dysregulation of nutritional metabolism (1). According to the statistics from the International Diabetes Federation, in 2019, there were 460 million diabetes patients worldwide, most of whom were T2D patients (2). Projections indicate that by 2030, T2D will impact 642 million people globally (3). Given the substantial prevalence of T2D and its associated complications, which has led to adverse social and economic impacts, it is crucial to delve into its pathogenesis and explore novel therapeutic targets.

Periodontitis (PD) is a chronic infectious disease characterized by gingival inflammation and destruction of periodontal tissue (4). As the seventh most prevalent disease worldwide, periodontitis has inflicted hundreds of millions of individuals, and there were 1.09 billion periodontitis cases by 2019 (5). In the USA, approximately 50% of adults aged 30 years or older have PD, and 49.4% of individuals in Japan suffer from PD. In addition to its detrimental impact on oral health, periodontitis also increases the risk of other diseases, such as diabetes, cardiovascular disease and Alzheimer’s disease (6). Furthermore, for PD patients suffer from T2D and other cardiovascular conditions, they may potentially experience the development of metabolic syndrome, which was characterized by hypertension, dyslipidemia, and central obesity (7). Therefore, the treatment of periodontitis confronts significant challenges, and it is extremely important to screen therapeutic targets for periodontitis and its associated complications to attain precise treatment.

Existing research indicates that periodontitis is a prevalent complication of T2D, and it also exerts a significant impact on the onset, progression and prognosis of T2D (8). Nascimento et al. demonstrated that diabetes increased the risk of occurrence or progression of periodontitis by 86% (RR=1.86, 95% CI: 1.30-2.80) (9). Furthermore, a meta-analysis of 53 observational studies revealed that severe periodontitis increased the incidence rate of T2D by 53%, and patients with T2D exhibited significantly worse periodontal condition (10). However, few studies provide profound insights into the treatment for patients with diabetes and periodontitis, let alone identify precise therapeutic targets. Due to the interference of confounding factors and reverse causal bias in traditional observational studies, the biomarkers proposed in previous studies cannot accurately guide the treatment of these two diseases. Currently, few studies have pinpointed precise genetic targets associated with the occurrence and progression of T2D as well as PD, and delved into their specific functions. Consequently, robust and novel analytical methods are needed to explore the therapeutic targets of T2D and periodontitis.

Single-cell RNA sequencing (scRNA-seq) technology, a revolutionary high-throughput sequencing method, enables the sequencing of individual cells, unraveling their heterogeneity and evolutionary relationship (11). In comparison to conventional sequencing techniques, scRNA-seq has the capability to identify rare cell types, unveiling the transcriptional regulatory networks and dynamic changes among cells (12). Currently, significant progression has been achieved in many fields such as microbiology, oncology and immunology with the help of scRNA-seq (13, 14). MR analysis is an epidemiological approach which has been widely applied to assess the causal association between exposure and outcome (15). Due to random allocation of genetic variants during conception, MR analysis can effectively mitigate the influence of confounding factors and reverse causal bias (16). Consequently, MR analysis is widely acknowledged for its exceptional capacity to identify novel therapeutic targets (17, 18). MR analysis is based on three key hypotheses: (1) Correlation hypothesis: The selected instrumental variables (IVs) must be robustly associated with the exposure; (2) Independence hypothesis: The IVs should not be associated with confounding factors of exposure and outcome; (3) Exclusivity hypothesis: The IVs exclusively influence the outcome through the exposure without any involvement of other ways (15). Furthermore, with the advent of expression quantitative trait loci (eQTLs) data, the causal association between genes and diseases could be investigated (19). At present, there is no study integrating both single-cell analysis and MR analysis to identify novel therapeutic targets for T2D and periodontitis, and only a small number of studies have employed single-cell analysis to interpret the association between T2D and periodontitis. However, these studies are superficial as the causal relationships remain unknown. Thus, their findings offer limited guidance for clinical applications.

**Figure 1:**
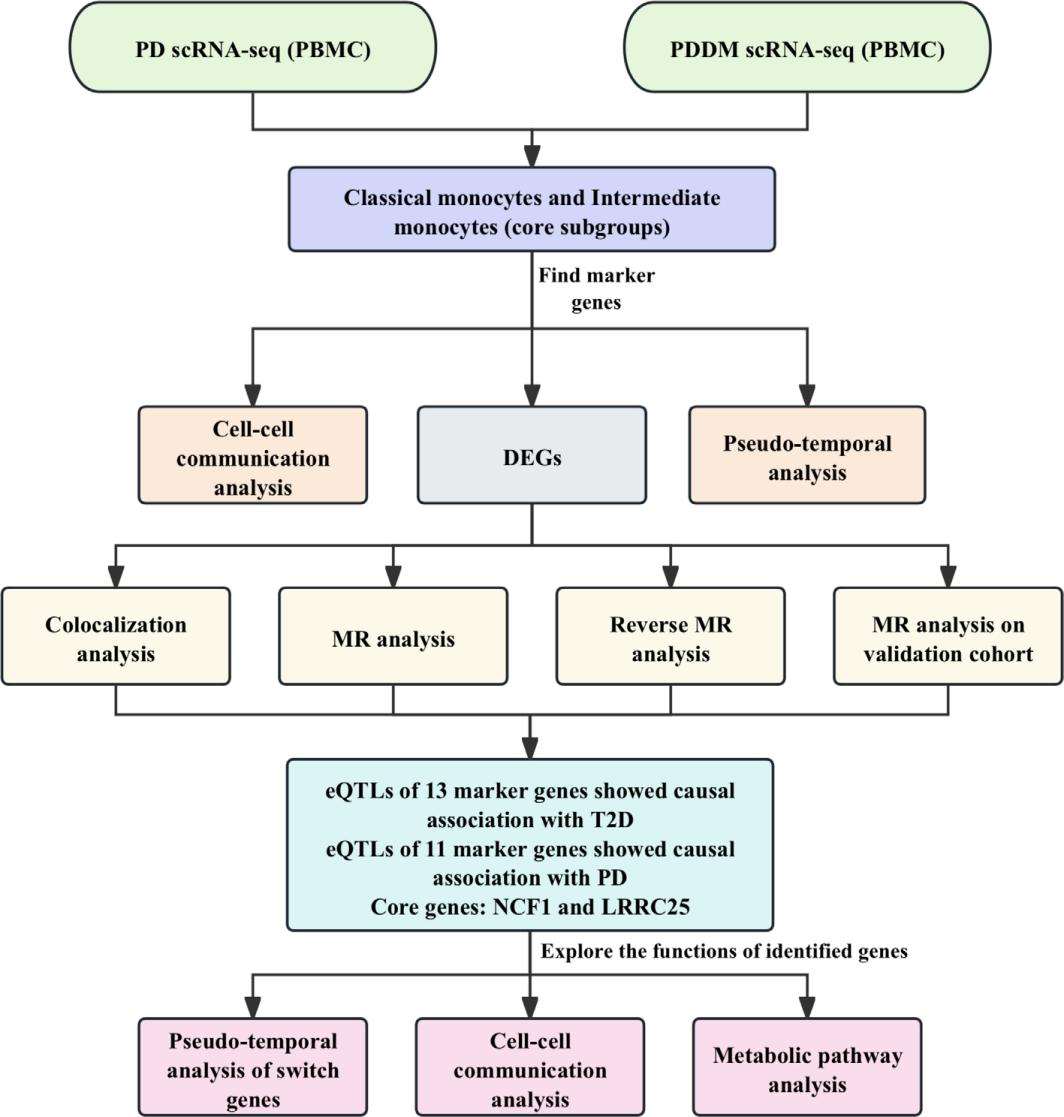
The workflow of this study. We integrated the scRNA-seq data from PD patients and PDDM patients for analysis. Preliminary analysis indicated that classical monocytes and intermediate monocytes were crucial cellular subgroups. Cell-cell communication and pseudo-temporal analyses were conducted and DEGs were identified. Subsequently, we conducted MR and colocalization analyses to explore the causal associations between identified genes and T2D as well as PD. Finally, NCF1 and LRRC25 were identified core genes, and we further explored the functions of the identified genes.

In this study, we aimed to integrate single-cell analysis and MR analysis to identify potential therapeutic targets for T2D and periodontitis, as well as validate their causal associations with these two diseases. Colocalization analysis, reverse MR analysis, additional validation and phenotype scanning further enhance the robustness of our findings. The discovery of these novel therapeutic targets holds promise for advancing the treatment and management of T2D and periodontitis. The detailed workflow of this study is illustrated in **Figure 1**.

## 2 Materials and methods

### 2.1 Data sources

The scRNA-seq data of peripheral blood mononuclear cells (PBMC) were acquired from the GEO database (https://www.ncbi.nlm.nih.gov/). Dataset GSE244515 provided 10X scRNA-seq data from patients with periodontitis, patients with periodontitis and T2D and healthy individuals (20). We selected data of two patients with periodontitis (GSM7818506 and GSM7818508), two patients with periodontitis and T2D (GSM7818516 and GSM7818517) and two healthy individuals (GSM7818496 and GSM7818497) for single-cell analysis. To evaluate the causal association between identified genes and diseases, we obtained blood expression quantitative trait loci (eQTLs) data of Differentially expressed genes (DEGs) from eQTLGen (https://www.eqtlgen.org/), which encompasses eQTLs data of 16,987 genes derived from 31,684 blood samples collected from healthy European population. During the target discovery phase, the GWAS data of T2D (29,193 cases and 182,573controls) and periodontitis (3,046 cases and 195,395 controls) from FinnGen consortium were utilized. As there was no available data on patients with both T2D and periodontitis, we selected the data of each disease respectively. For additional validation, we obtained GWAS data of T2D from IEU Open GWAS (https://gwas.mrcieu.ac.uk). The GWAS ID of the summary data of T2D was ebi-a-GCST90018926 (38,841 cases and 451,248 controls) (21). Unfortunately, we were unable to find additional high-quality GWAS data for periodontitis. Consequently, we did not carry out additional validation on this part of the findings. For MR analysis, there was no overlap between the exposure and outcome.

### 2.2 Single-cell data processing and analysis

For single-cell analysis, we utilized R package “Seurat” (V.4.3.0) for data processing and visualization (22). During the quality control process, we excluded cells with less than 200 or more than 4,000 feature genes. Besides, Cells with more than 10% mitochondrial genes were also removed. Subsequently, data normalization was performed to mitigate batch effects and 2,000 highly variable genes (HVGs) were identified for principal component analysis (PCA). Furthermore, we employed R package “harmony” (V.0.1.1) to further alleviate the impact of batch effects (23). Next, we employed the Uniform Manifold Approximation and Projection (UMAP) method to reduce data dimensionality and perform cell clustering. Cell types of each cluster were annotated using R package “singleR” (V.2.2.0) based on data from Human Primary Cell Atlas (24). At last, by calculating the proportion of each cell cluster and integrating our findings with existing literature reports, we identified monocytes as an important cell subcluster. Subsequent analysis was then carried out specifically focusing on this subcluster. The analysis strategies for this subcluster were similar to the aforementioned steps.

### 2.3 Pseudo-temporal analysis and cell-cell communication analysis

With the help of package “slingshot” (V.2.10.0) and “SingleCellExperiment” (V.1.20.0), we conducted cell trajectory analysis for monocytes clusters (25, 26). To obtain comprehensive insights into the functions and roles of the core cell clusters, we performed cell-cell communication analysis using the R package “CellChat” (V.1.6.1) (27). We analyzed the interactions among different cell clusters and identified the ligand-receptor pairs. Function “netVisual_circle” and “pp_bubble” were used to visualize the results.

### 2.4 Differentially expressed genes in the core subclusters

At last, classical monocytes and intermediate monocytes were identified as core cell subclusters due to their large proportion. Subsequently, we employed “FindMarkers” function of “Seurat” package, setting the log fold change threshold at 0.5. We identified DEGs in classical monocytes and intermediate monocytes with other monocytes, as well as with non- monocytes, separately. After intersecting the sets of DEGs, we obtained two distinct gene sets that exclusively representing uniquely expressed genes in classical monocytes and intermediate monocytes. Furthermore, we conducted enrichment analysis using “Metascape” (V.3.5) to unveil the functional significance of these DEGs (28).

### 2.5 Identification of IVs and MR analysis

We employed R package “TwoSampleMR” (V.0.5.8) for MR analysis to evaluate the causal association among DEGs, T2D and periodontitis. Single Nucleotide Polymorphisms (SNPs) that satisfied the aforementioned three hypotheses were chosen as instrumental variables (IVs). Firstly, the IVs should reach the threshold of genome-wide significance (*p* < 5 $\;\times 10^{-8}$ ). Secondly, as the IVs must be independent, we conducted clumping (kb = 10,000, $r^{2}$ < 0.001) to avoid linkage disequilibrium. Furthermore, we calculated the $R^{2}$ value and F-statistics of each SNP to avoid weak instrument bias. SNPs with F-statistics less than 10 were considered weak IVs and were excluded (29). At last, palindromic SNPs and SNPs containing missing data were removed.

In the primary MR analysis, data of gene eQTLs were used as exposures, and GWAS data of T2D and periodontitis were used as outcomes. If the gene had only one IV, Wald ratio method was employed to generate causal effect estimate (30). In contrast, if there were two or more IVs available, we used inverse-variance weighted (IVW) method as the primary method (31). To make our findings more robust, MR-Egger method, Weighted Median method and Weighted Mode method were also applied (32–34). As we know, IVW exhibits the most efficient with the greatest statistical power, which is not affected by horizontal pleiotropy when all IVs are valid (Satisfy the three hypotheses) (31). Other methods have a higher tolerance of invalid IVs, which can generate relatively credible causal estimates when not all IVs are valid (32–34). Besides, sensitivity analysis was conducted. Heterogeneity was assessed through Cochran’s Q test and horizontal pleiotropy was detected by MR-Egger intercept test. *P*-value > 0.05 indicated the absence of significant heterogeneity and horizontal pleiotropy (32, 35). Causal genes for T2D or periodontitis were identified based on the following criteria: (1) The *p*-value of IVW method or Wald ratio method < 0.05. (2) Significant heterogeneity and horizontal pleiotropy did not exist. (3) The directionality of IVW method was consistent with other three methods (There were 3 or more IVs available).

### 2.6 Colocalization analysis

To further validate our findings in MR analysis, we performed Bayesian colocalization analysis to investigate whether the identified causal associations were driven by linkage disequilibrium (30). R package “coloc” (V.5.2.3) were employed with the following settings: (1) $P_{1}$ =1 $\;\times 10^{-4}$ : The prior probability of the SNP being exclusively associated with trait 1; (2) $P_{2}$ =1 $\;\times 10^{-4}$ : The probability of the SNP being exclusively associated with trait 2; (3) $P_{12}$ =1 $\;\times 10^{-5}$ : The probability of the SNP being associated with both traits (36). In addition, in colocalization analysis, posterior probabilities (PP) of 5 hypotheses were assessed: (1) H0: Both trait 1 and trait 2 do not have causal SNP; (2) H1: Only trait 1 has a causal SNP; (3) H2: Only trait 2 has a causal SNP; (4) H3: Both trait 1 and trait 2 have a causal SNP, but the causal variants are distinct; (5) H4: Both trait 1 and trait 2 have a causal SNP, and they share the same SNP (37). Substantial evidence of colocalization was identified at PPH4 > 0.8 (38). Besides, medium evidence of colocalization was identified at PPH3+PPH4 > 0.8. (Both exposure and outcome have a causal SNP) (39). We used R package “LocusCompareR” (V.1.0.0) for the visualization of results of colocalization analysis (40).

### 2.7 Phenotype scanning

Employing “LDTrait” tool (41), we conducted phenotype scanning to explore the associations of IVs of identified causal genes with other diseases and traits. The causal genes were considered to exhibit pleiotropic effects when they satisfy the following criteria: (1) IVs of genes reached the genome-wide significant threshold (*p* < 5 $\;\times 10^{-8}$ ). (2) IVs of genes exhibited significant association either directly with the two diseases or with well-established risk factors of the two diseases.

### 2.8 Reverse MR analysis and additional validation

To bolster the reliability of our findings, we employed aforementioned strategies to obtain IVs of T2D and periodontitis. Then, reverse MR analysis was carried out to explore the potential instances of reverse causality. In this analysis, GWAS data of T2D and periodontitis were used as exposure, and gene eQTLs data were used as outcome. A *p*-value of Wald ratio or IVW method < 0.05 indicated the presence of reverse causality.

Given that NCF1 and LRRC25 were identified as core therapeutic targets for T2D and periodontitis, we further validated the causal associations in another cohort using the same analysis strategies mentioned in 2.5.

### 2.9 Exploring the functions of identified genes

We further analyzed the cell clusters based on the identified causal genes to preliminarily explore the underlying mechanisms of observed causal relationships. Firstly, we employed function “DotPlot” to visualize the expression of 23 causal genes across different cell clusters. Additionally, causal genes were further analyzed using R package “GeneSwitches” (V.0.1.0). Switch genes that might play crucial roles in trajectory development were identified for conducting pseudo-temporal differentiation trajectory analysis, and we depicted the gene expression patterns over pseudo-time (42). In order to highlight the expression of core genes (NCF1 and LRRC25) over pseudo-time, we utilized function “ggscatterstats”.

Based on the expression of two core causal genes (NCF1 and LRRC25), the core subcluster was divided into two clusters (expressing or not expressing the marker genes). Using the package “CellChat”, we analyzed the intercellular communications among different cell clusters and identified the ligand-receptor pairs (27). At last, Metabolic pathway analysis was conducted using the package “scMetabolism” (V.0.2.1) with parameters “method = VISION, metabolism.type = KEGG” (43).

### 2.10 Statistical analysis

In this study, standard statistical tests including Student’s t-test, Wilcoxon rank-sum test, Kruskal-Wallis test and Chi-square test were employed to evaluate differences among various cell subclusters. All the statistical analyses were conducted using R 4.1.3, and threshold of *p* < 0.05 was considered statistically significant.

## 3 Results

### 3.1 Analysis of scRNA-seq data

**Figure 2:**
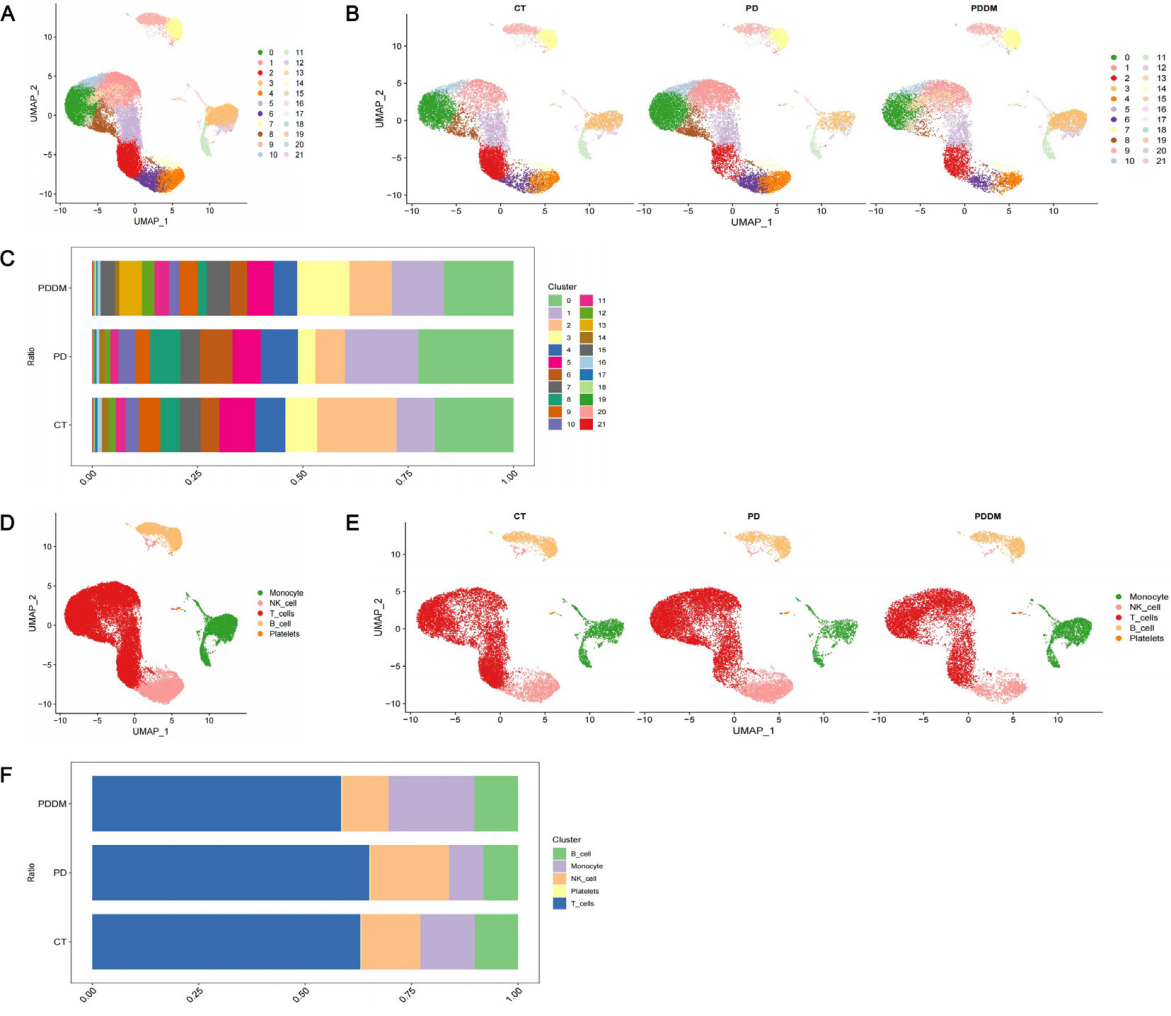
Single-cell transcriptional profiling. **(A)** UMAP plot of identified cell clusters (before annotation); **(B)** UMAP plot of identified cell clusters in PD patients, PDDM patients and healthy individuals, respectively (before annotation); **(C)** The proportion of each subcluster in PD patients, PDDM patients and healthy individuals (before annotation); **(D)** UMAP plot of identified cell clusters (after annotation); **(E)** UMAP plot of identified cell clusters in PD patients, PDDM patients and healthy individuals, respectively (after annotation); **(F)** The proportion of each subcluster in PD patients, PDDM patients and healthy individuals (after annotation). PD, Periodontitis; PDDM, Periodontitis and type II diabetes.

**Figure 3:**
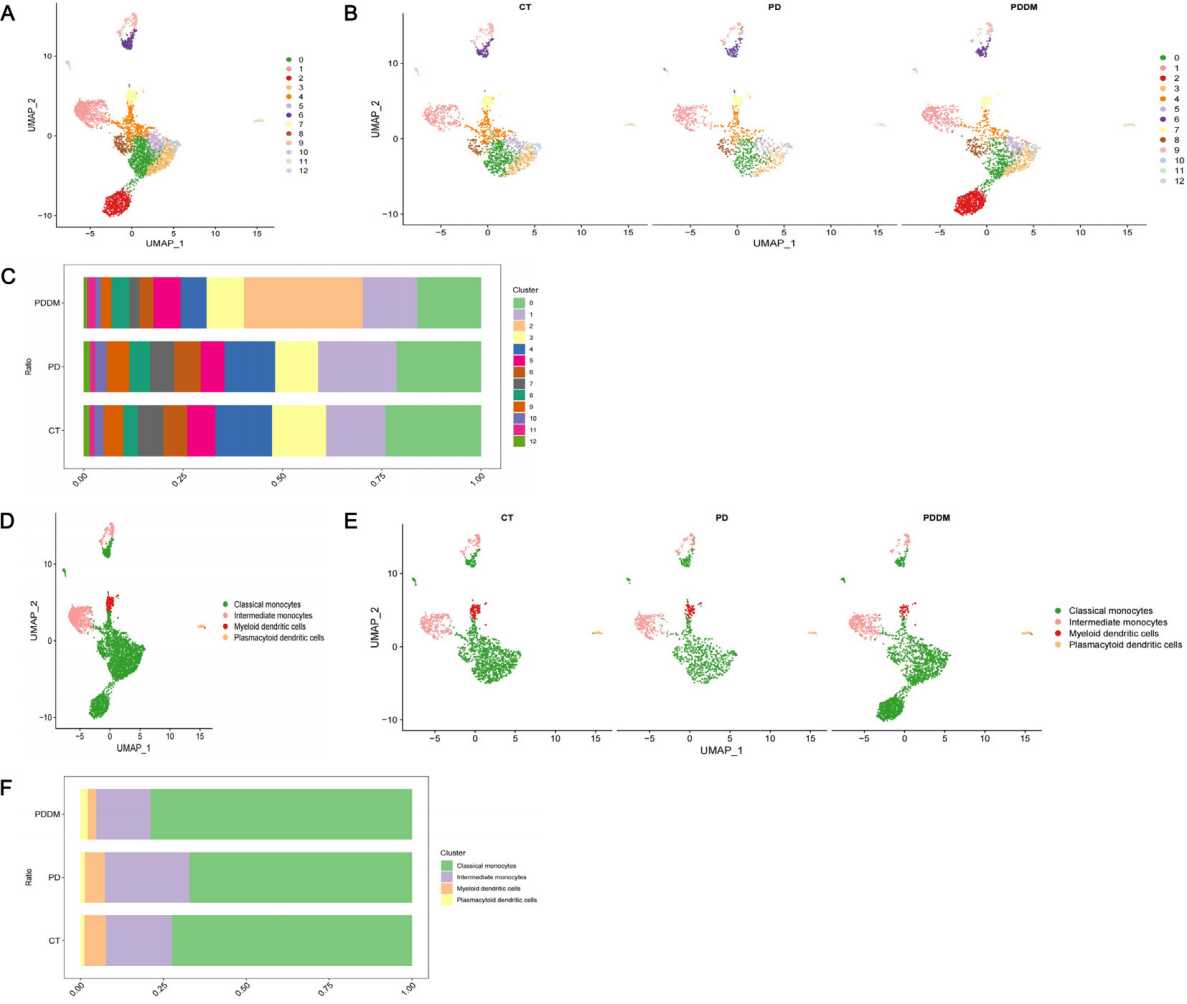
In-depth analysis and visualization of monocytes. **(A)** UMAP plot of identified cell clusters in monocytes (before annotation); **(B)** UMAP plot of identified cell clusters in monocytes in PD patients, PDDM patients and healthy individuals, respectively (before annotation); **(C)** The proportion of each subcluster in monocytes in PD patients, PDDM patients and healthy individuals (before annotation); **(D)** UMAP plot of identified cell clusters in monocytes (after annotation); **(E)** UMAP plot of identified cell clusters in PD patients, PDDM patients and healthy individuals, respectively (after annotation); **(F)** The proportion of each subcluster of monocytes in PD patients, PDDM patients and healthy individuals (after annotation). PD, Periodontitis; PDDM, Periodontitis and type II diabetes.

Through analyzing scRNA-seq data, 22 cell clusters were identified (**Figures 2A, B**). After annotation, the 22 cell clusters were classified into 5 clusters: Monocytes, Natural Killer (NK) cells, T cells, B cells and Platelets (**Figures 2D, E**). The proportion of each cell clusters are presented in **Figures 2C, F**. Compared with healthy individuals, it was noted that the proportion of monocytes in patients with periodontitis exhibited a significant decrease, while the proportion of monocytes in patients with diabetes and periodontitis was increased (**Figure 2F**). Existing research has also demonstrated the crucial roles of monocytes in patients with diabetes and periodontitis (44–46). Especially Shen et al. proposed a treatment strategy to alleviate diabetes related periodontitis through inhibiting inflammatory monocyte infiltration (44). Consequently, monocytes were identified as the important cell cluster, and we extracted scRNA-seq data of monocytes for further analysis. Within monocyte cluster, we initially identified 13 cell clusters (**Figures 3A, B**). After annotation, the 13 cell clusters were classified into 4 clusters: classical monocytes, intermediate monocytes, myeloid dendritic cells and plasmacytoid dendritic cells (**Figures 3D, E**). The proportion of each cell clusters are presented in **Figures 3C, F**. In monocytes, Classical monocytes and Intermediate monocytes accounted for the vast majority (**Figure 3F**). As a result, they were regarded as the core subclusters for further in-depth analysis.

### 3.2 Cell trajectory analysis and cell-cell communication analysis

**Figure 4:**
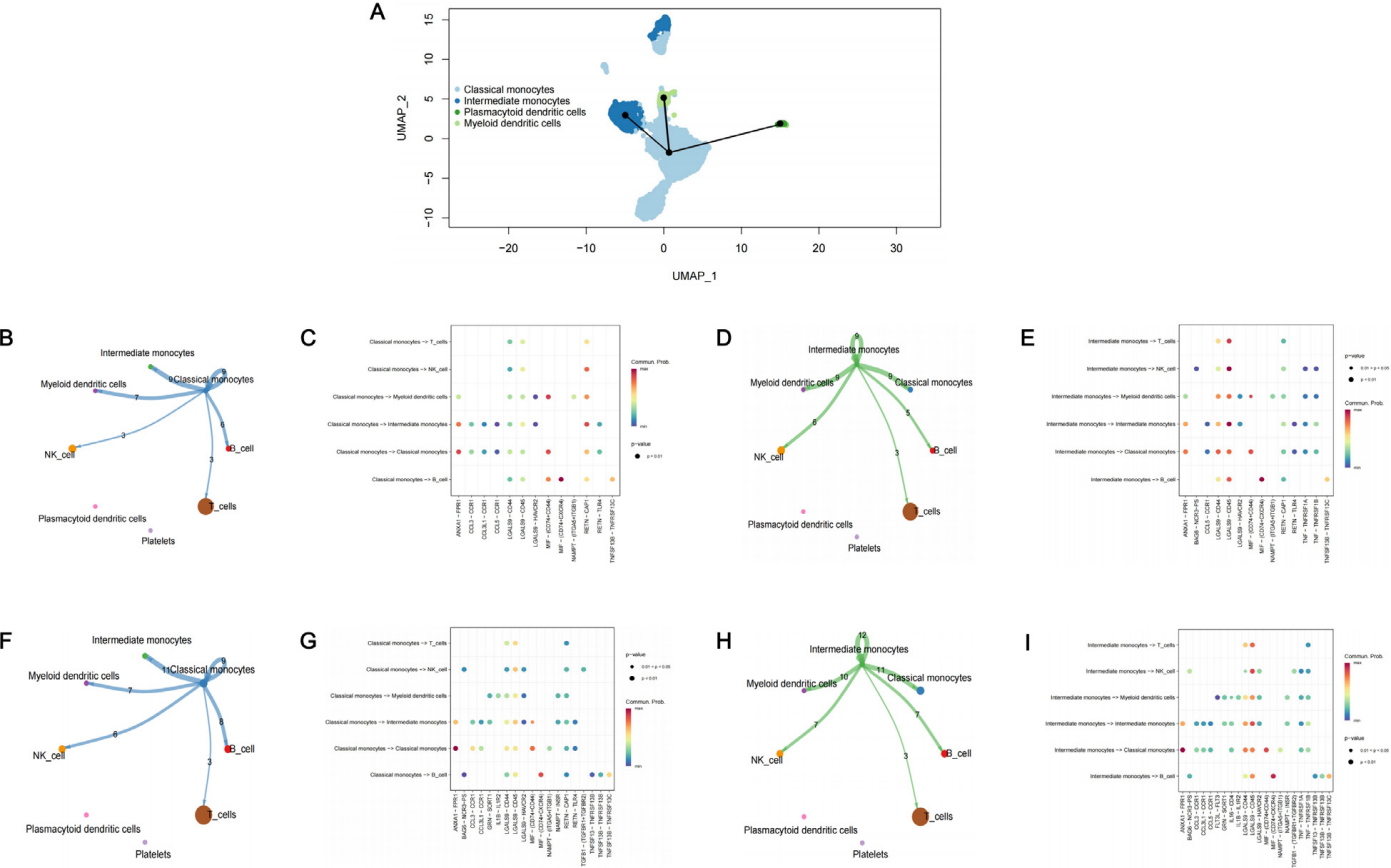
Cell trajectory analysis and cell-cell communication analysis. **(A)** The cell trajectory analysis of monocytes; **(B)** Cell-cell communication analysis in PD patients among classical monocytes and other monocytes; **(C)** The bubble plot of possible interactions in PD patients among classical monocytes and other monocytes; **(D)** Cell-cell communication analysis in PD patients among intermediate monocytes and other monocytes; **(E)** The bubble plot of possible interactions in PD patients among intermediate monocytes and other monocytes; **(F)** Cell-cell communication analysis in PDDM patients among classical monocytes and other monocytes; **(G)** The bubble plot of possible interactions in PDDM patients among classical monocytes and other monocytes; **(H)** Cell-cell communication analysis in PDDM patients among intermediate monocytes and other monocytes; **(I)** The bubble plot of possible interactions in PDDM patients among intermediate monocytes and other monocytes; PD, Periodontitis; PDDM, Periodontitis and type II diabetes.

Through pseudo-temporal analysis, we identified the differentiation trajectory of monocytes (**Figure 4A**). Monocytes originated from classical monocytes and underwent differentiation into intermediate monocytes, plasmacytoid dendritic cells and myeloid dendritic cells. As classical monocytes and intermediate monocytes were identified as core cell subclusters, we conducted cell-cell communication analysis in patients only with periodontitis and patients with both diabetes and periodontitis to explore the interactions among core cell subclusters and other cell subclusters. Both classical monocytes and intermediate monocytes were found to interacted with other cell clusters except for platelets and plasmacytoid dendritic cells, mainly through pathways associated with LGALS9 and RETN (**Figures 4B–I**).

### 3.3 MR analysis between DEGs and diseases

**Figure 5:**
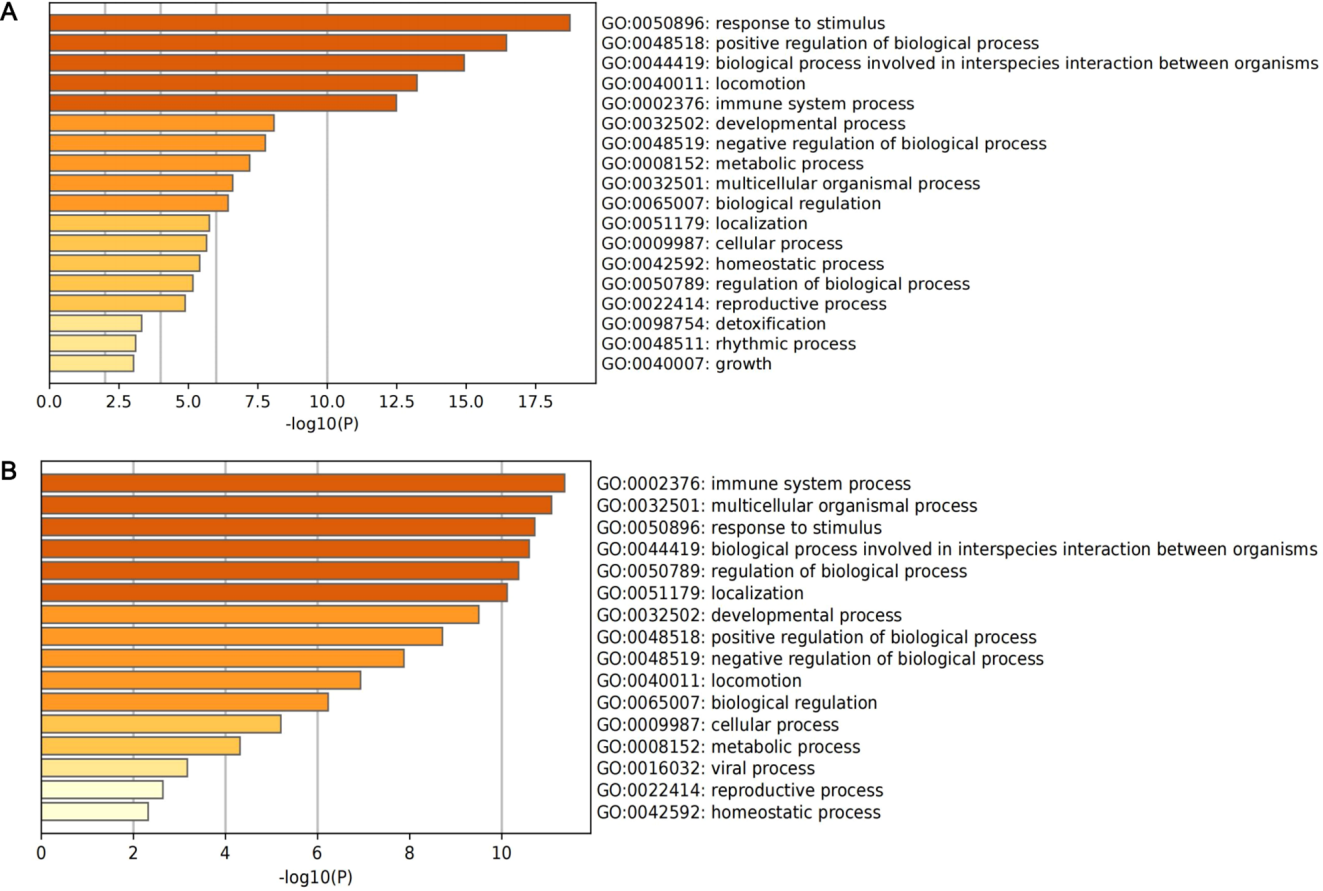
The histogram of enrichment analysis based on DEGs of core subclusters. **(A)** Enrichment analysis of 127 DEGs of classical monocytes; **(B)** enrichment analysis of 94 DEGs of intermediate monocytes.

A total of 127 DEGs of classical monocytes and 94 DEGs of intermediate monocytes were identified as marker genes (**Supplementary Table S1**). The results of enrichment analysis are presented in **Figure 5**. For classical monocytes, DEGs were mainly enriched in terms of response to stimulus and positive regulation of biological process. For intermediate monocytes, DEGs were mainly enriched in immune system process, multicellular organismal process and response to stimulus.

**Figure 6:**
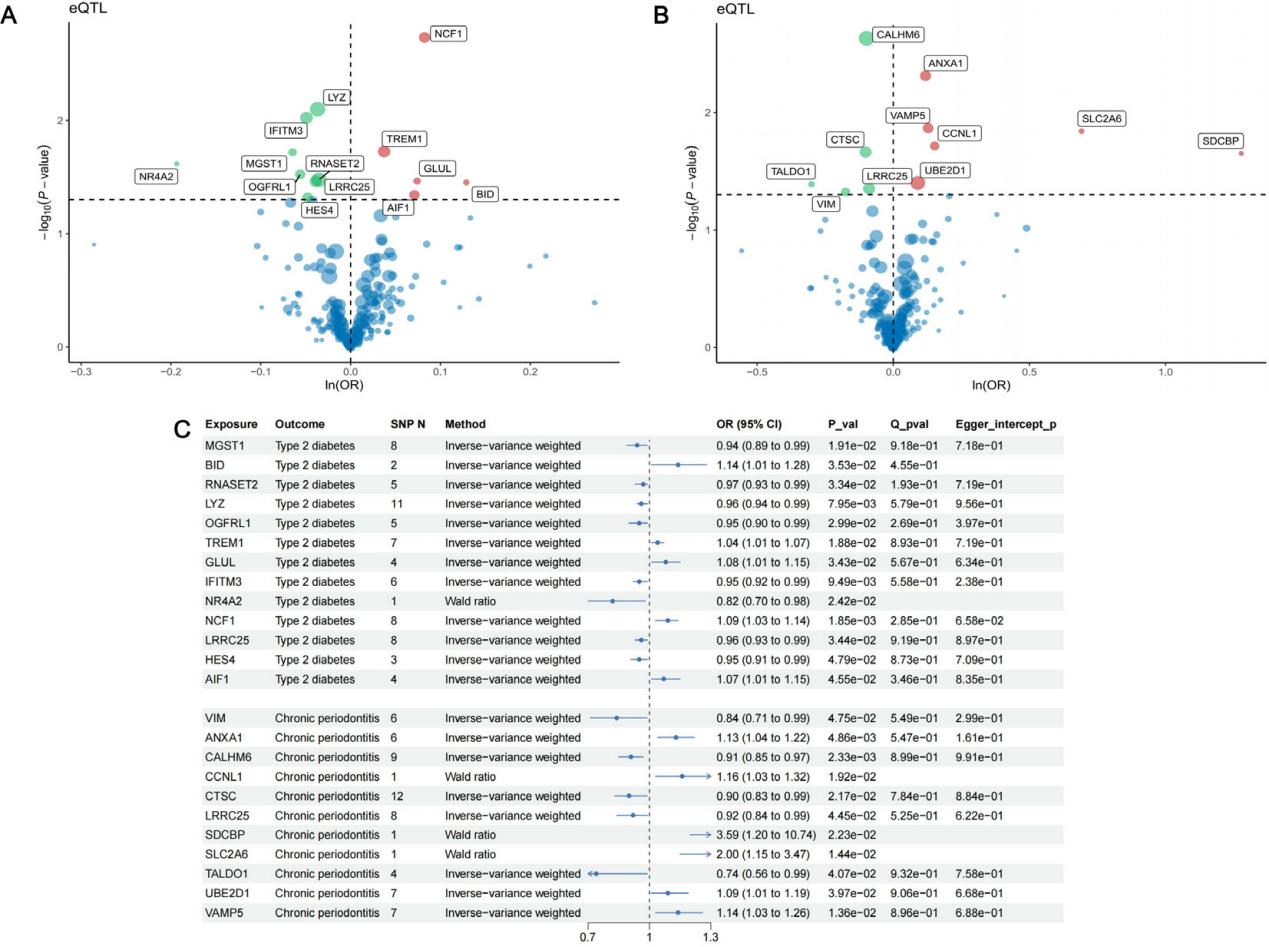
Results of MR analysis. **(A)** The volcano plot of the results of MR analysis between 221 DEGs and type II diabetes; **(B)** The volcano plot of the results of MR analysis between 221 DEGs and periodontitis. The horizontal line indicates a *p*-value of 0.05. Red dots represent risk genes of the disease, green dots represent protective genes of the disease, and blue dots represent Neutral genes of the disease. **(C)** Forest plot of MR analysis between 13 identified causal genes and type II diabetes, 11 causal genes with periodontitis.

Finally, 1056 SNPs were selected as IVs for the 221 identified marker genes. The F-statistics of IVs ranged from 29.76 to 4766.89, indicating that there was no weak instrument bias (**Supplementary Table S2**). In the MR analysis with T2D as outcome, 13 genes presented causal association with T2D (**Figure 6**): MGST1 (OR = 0.94, 95% CI: 0.89-0.99, *p* = 1.91 $\;\times 10^{-2}$ ), BID (OR = 1.14, 95% CI: 1.01-1.28, *p* = 3.53 $\;\times 10^{-2}$ ), RNASET2 (OR = 0.97, 95% CI: 0.93-0.99, *p* = 3.34 $\;\times 10^{-2}$ ), LYZ (OR = 0.96, 95% CI: 0.94-0.99, *p* = 7.95 $\;\times 10^{-3}$ ), OGFRL1 (OR = 0.95, 95% CI: 0.90-0.99, *p* = 2.99 $\;\times 10^{-2}$ ), TREM1 (OR = 1.04, 95% CI: 1.01-1.07, *p* = 1.88 $\;\times 10^{-2}$ ), GLUL (OR = 1.08, 95% CI: 1.01-1.15, *p* = 3.43 $\;\times 10^{-2}$ ), IFITM3 (OR = 0.95, 95% CI: 0.92-0.99, *p* = 9.49 $\;\times 10^{-3}$ ), NR4A2 (OR = 0.82, 95% CI: 0.70-0.98, *p* = 2.42 $\;\times 10^{-2}$ ), NCF1 (OR = 1.09, 95% CI: 1.03-1.14, *p* = 1.85 $\;\times 10^{-3}$ ), LRRC25 (OR = 0.96, 95% CI: 0.93-0.99, *p* = 3.44 $\;\times 10^{-2}$ ), HES4 (OR = 0.95, 95% CI: 0.91-0.99, *p* = 4.79 $\;\times 10^{-2}$ ) and AIF1 (OR = 1.07, 95% CI: 1.01-1.15, *p* = 4.55 $\;\times 10^{-2}$ ). In the MR analysis with periodontitis as outcome, we identified 11 causal genes (**Figure 6**): VIM (OR = 0.84, 95% CI: 0.71-0.99, *p* = 4.75 $\;\times 10^{-2}$ ), ANXA1 (OR = 1.13, 95% CI: 1.04-1.22, *p* = 4.86 $\;\times 10^{-3}$ ), CALHM6 (OR = 0.91, 95% CI: 0.85-0.97, *p* = 2.33 $\;\times 10^{-3}$ ), CCNL1 (OR = 1.16, 95% CI: 1.03-1.32, *p* = 1.92 $\;\times 10^{-2}$ ), CTSC (OR = 0.90, 95% CI: 0.83-0.99, *p* = 2.17 $\;\times 10^{-2}$ ), LRRC25 (OR = 0.92, 95% CI: 0.84-0.99, *p* = 4.45 $\;\times 10^{-2}$ ), SDCBP (OR = 3.59, 95% CI: 1.20-10.74, *p* = 2.23 $\;\times 10^{-2}$ ), SLC2A6 (OR = 2.00, 95% CI: 1.15-3.47, *p* = 1.44 $\;\times 10^{-2}$ ), TALDO1 (OR = 0.74, 95% CI: 0.56-0.99, *p* = 4.07 $\;\times 10^{-2}$ ), UBE2D1 (OR = 1.09, 95% CI: 1.01-1.19, *p* = 3.97 $\;\times 10^{-2}$ ) and VAMP5 (OR = 1.14, 95% CI: 1.03-1.26, *p* = 1.36 $\;\times 10^{-2}$ ). Cochran’s Q test and MR-Egger intercept test failed to find significant heterogeneity and horizontal pleiotropy (**Figure 6**). Detailed information of MR analysis is presented in **Supplementary Tables S3**-**S5**.

Among the 13 causal genes of T2D, there were 5 risk genes and 8 protective genes. For periodontitis, 6 genes presented risk association and 5 genes were identified as protective genes. It should be noted that LRRC25 was identified as protective genes for both T2D (OR = 0.96, 95% CI: 0.93-0.99, *p* = 3.44 $\;\times 10^{-2}$ ) and periodontitis (OR = 0.92, 95% CI: 0.84-0.99, *p* = 4.45 $\;\times 10^{-2}$ ). This finding suggests that LRRC25 holds promise as significant therapeutic target for T2D and periodontitis.

To examine whether reverse causal association existed, we performed reverse MR analysis. For the 13 causal genes of T2D, the results of reverse MR analysis demonstrated that there was no reverse causal relationship (**Supplementary Table S6**). For the 11 causal genes of periodontitis, unfortunately, there were no SNP left after extracting outcome data. As a result, we could not conduct reverse MR analysis between periodontitis and 11 causal genes. In this case, we assumed that reverse causal association did not exist.

### 3.4 Colocalization analysis, additional validation and phenotype scanning

**Figure 7:**
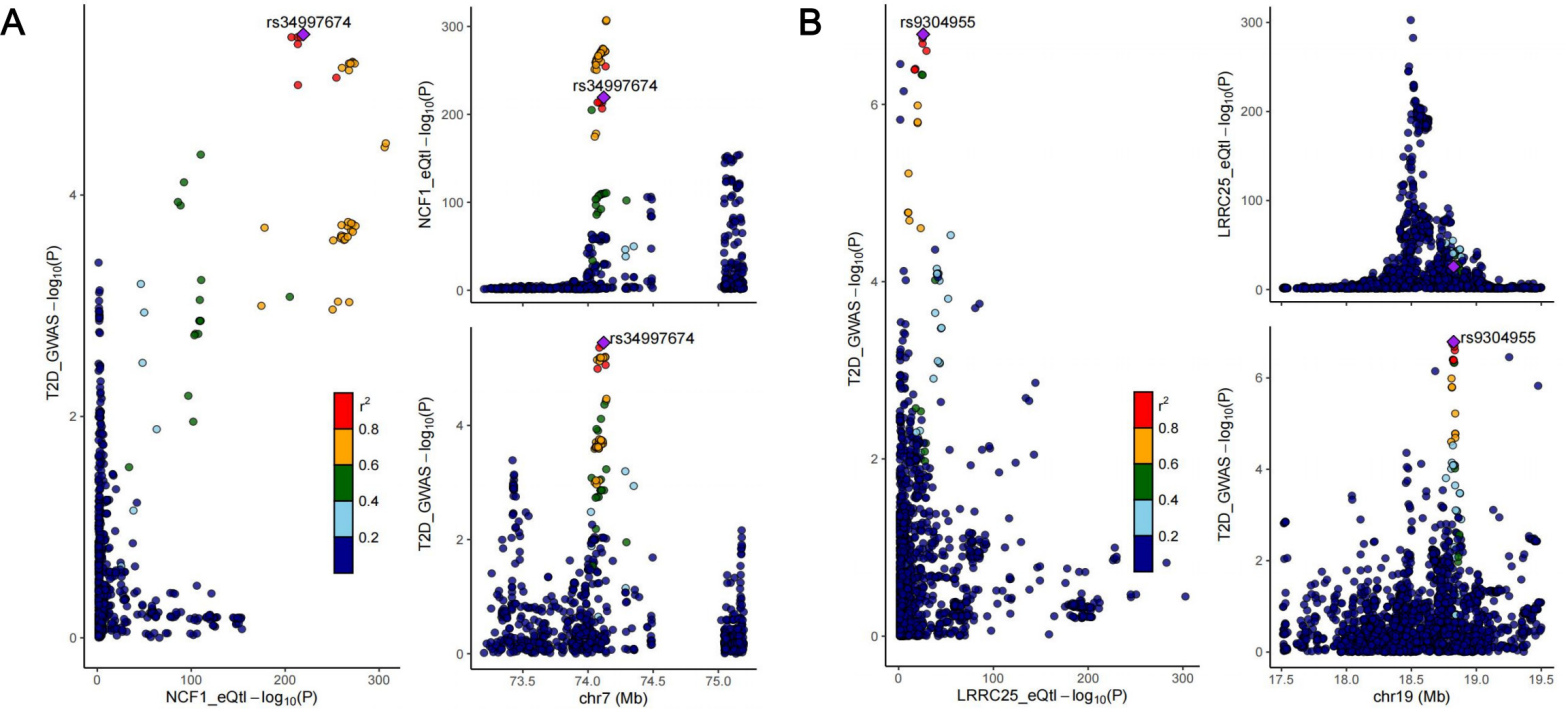
Regional association plots of colocalization analysis between NCF1 and type II diabetes **(A)**; LRRC25 and type II diabetes **(B)**.

We conducted colocalization analysis between the identified causal genes and the two diseases to explore shared genetic signals. Finally, NCF1 exhibited colocalization with T2D (PPH4 = 0.908), indicating that NCF1 and T2D shared the same causal genetic variant. Besides, median colocalization evidence was identified between LRRC25 and T2D (PPH3 + PPH4 = 0.992). Consequently, we identified NCF1 as core therapeutic targets for T2D and LRRC25 for T2D and periodontitis. The regional association plots between NCF1, LRRC25 and T2D are presented in **Figure 7**. Detailed results of colocalization analysis are presented in **Supplementary Table S7**.

**Figure 8:**
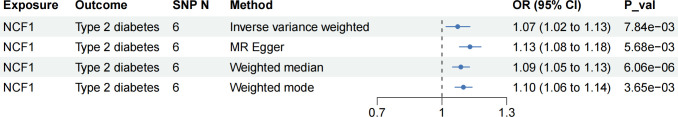
Forest plot of MR analysis between NCF1 and type II diabetes (In the validation cohort).

To enhance the robustness of our findings, we utilized data from another cohort as outcome to validate the observed causal relationships. Regrettably, we did not find additional high-quality data of periodontitis, so we only validated the causal relationship between NCF1, LRRC25 and T2D. In the validation cohort, NCF1 still presented risk causal association with T2D (OR = 1.07, 95% CI: 1.02-1.13, *p* = 7.84 $\;\times 10^{-3}$ ), and the direction of IVW method was consistent with other three methods (**Figure 8**). However, we did not observe the causal association between LRRC25 and T2D in this cohort (**Supplementary Table S8**).

In the phenotype scanning phase, we employed “LDTrait” tool to identify whether the IVs of the 23 causal genes were associated with T2D, periodontitis and their risk factors. The detailed results of phenotype scanning are presented in **Supplementary Tables S9**, **S10**. None of the SNP was directly associated with T2D or periodontitis. For the two core genes (NCF1 and LRRC25), IVs of them were mainly associated with content and proportion of blood cell. These findings further eliminated potential pleiotropy and enhanced the reliability of our results.

### 3.5 Explore the functions of identified targets

To preliminarily interpret the observed causal associations, we analyzed the expression of genes, intercellular communications and metabolic pathway activity within different cell clusters.

**Figure 9:**
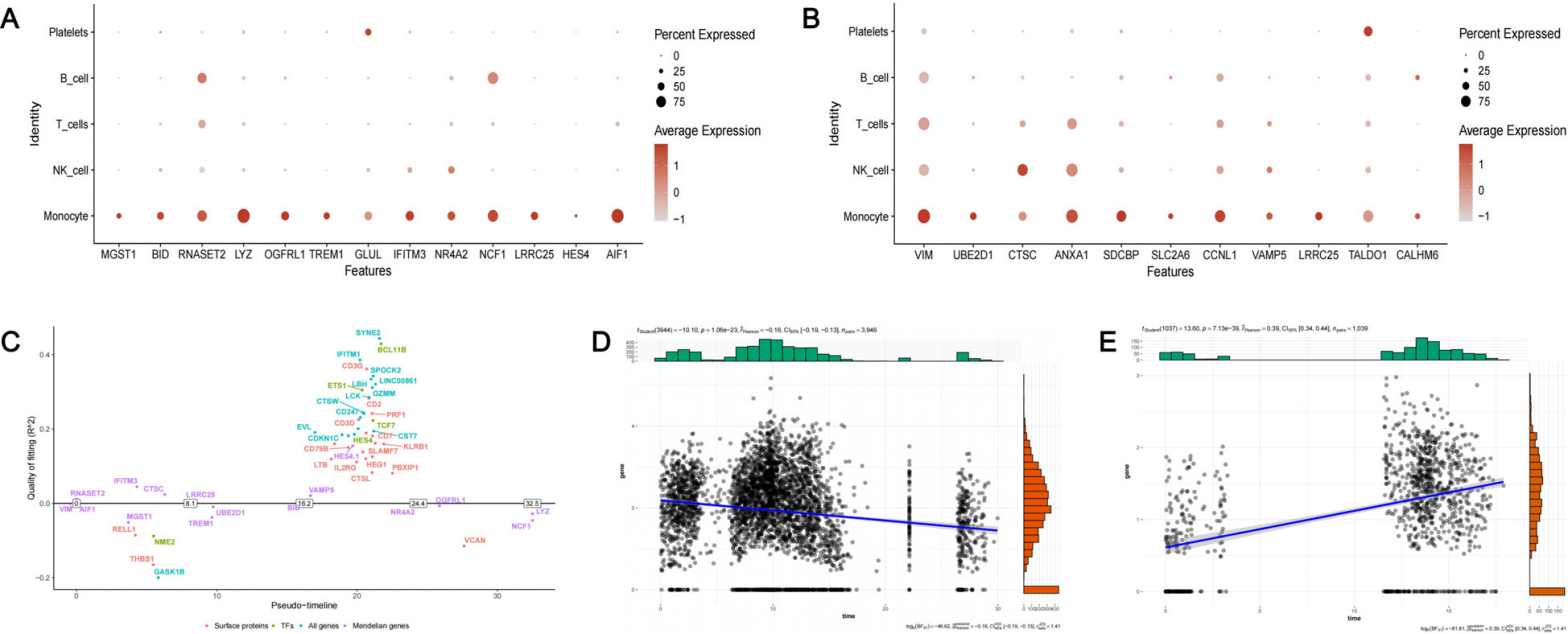
The expression of identified causal genes in cells. **(A)** The expression patterns of 13 causal genes of T2D in PDDM patients; **(B)** The expression patterns of 11 causal genes of periodontitis in PD patients; **(C)** The pseudo-temporal differentiation trajectory analysis of switch genes; **(D)** Scatter plot depicting the relationship between the expression of NCF1 and pseudo-time; **(E)** Scatter plot depicting the relationship between the expression of LRRC25 and pseudo-time.

We firstly visualized the expression patterns of 23 causal genes across different cell clusters (**Figures 9A, B**). **Figure 9A** depicted the expression patterns of 13 causal genes of T2D in patients with T2D and periodontitis, and **Figure 9B** depicted the expression patterns of 11 causal genes of periodontitis in patients with periodontitis. The causal genes exhibited high expression in monocytes. In addition, RNASET2 and NCF1 were also highly expressed in B cells. GLUL and TALDO1 were also highly expressed in platelets. Several genes such as VIM, CTSC, ANXA1, and CCNL1 have certain expression levels in multiple cell clusters. Furthermore, we depicted the states of switch genes over pseudo-time to investigate the changes in gene expression during cell development process (**Figure 9C**). For the two core genes (NCF1 and LRRC25), we employed scatter plots to illustrate the association between their expression and pseudo-time. The results indicated that the expression of NCF1 might decrease (Pearson *r* = -0.16, *p* < 0.001) and the expression of LRRC25 might increase (Pearson *r* = 0.39, *p* < 0.001) over pseudo-time (**Figures 9D, E**).

**Figure 10:**
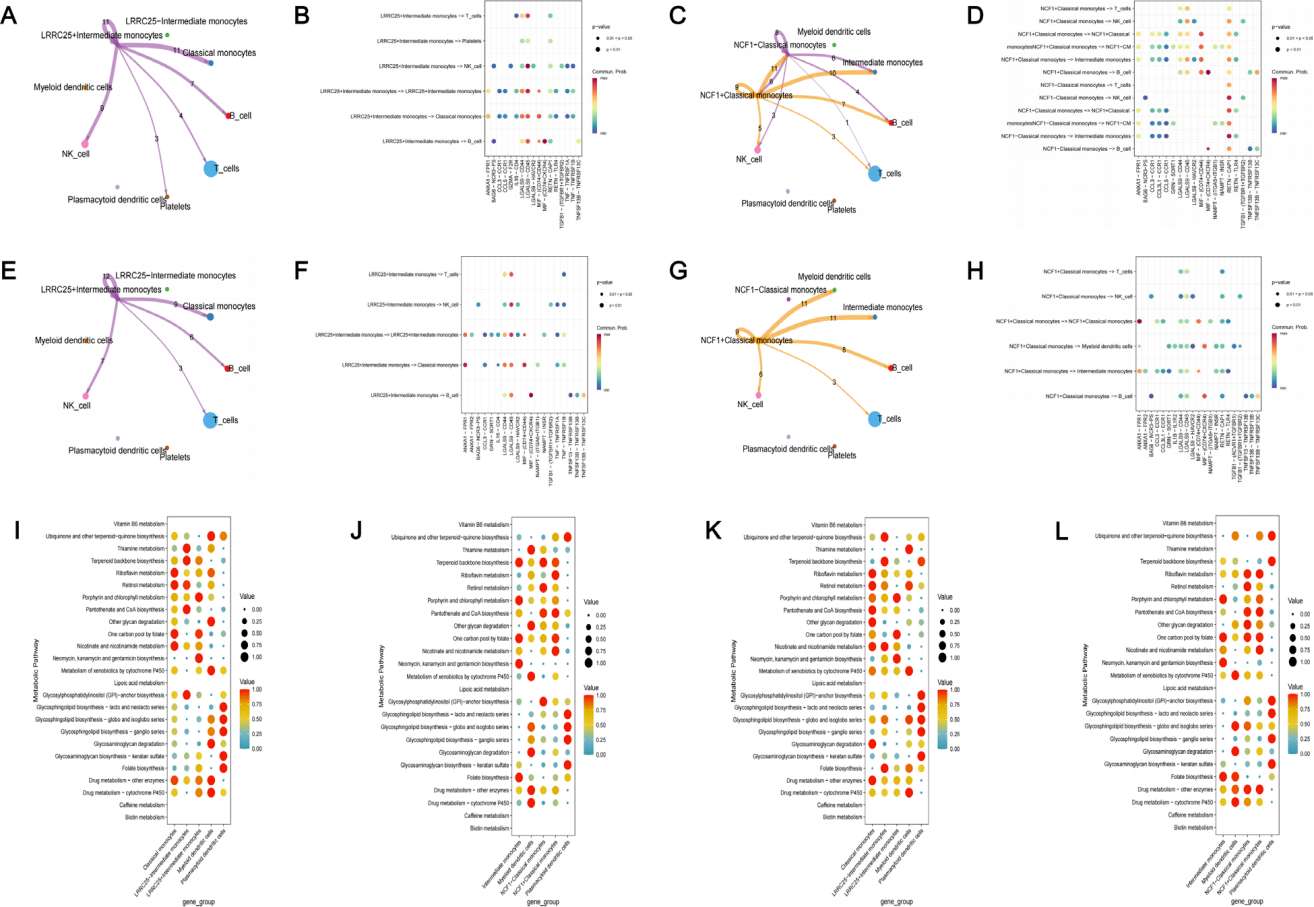
Functional analysis of identified targets. **(A)** Cell-cell communication analysis in PD patients among LRRC25^+^ IM, LRRC25^–^ IM and other monocytes; **(B)** The bubble plot of possible interactions among LRRC25^+^ IM, LRRC25^–^ IM and other monocytes in PD patients; **(C)** Cell-cell communication analysis in PD patients among NCF1^+^ CM, NCF1^–^ CM and other monocytes; **(D)** The bubble plot of possible interactions among NCF1^+^ CM, NCF1^–^ CM and other monocytes in PD patients; **(E)** Cell-cell communication analysis in PDDM patients among LRRC25 ^+^IM, LRRC25^–^ IM and other monocytes; **(F)** The bubble plot of possible interactions among LRRC25^+^ IM, LRRC25^–^ IM and other monocytes in PDDM patients; **(G)** Cell-cell communication analysis in PDDM patients among NCF1^+^ CM, NCF1^–^ CM and other monocytes; **(H)** The bubble plot of possible interactions among NCF1^+^ CM, NCF1^–^ CM and other monocytes in PDDM patients; **(I)** The activity of metabolic pathway of LRRC25^+^ IM, LRRC25^–^ IM and other monocytes in PD patients; **(J)** The activity of metabolic pathway of NCF1^+^ CM, NCF1^–^ CM and other monocytes in PD patients; **(K)** The activity of metabolic pathway of LRRC25^+^ IM, LRRC25^–^ IM and other monocytes in PDDM patients; **(L)** The activity of metabolic pathway of NCF1^+^ CM, NCF1^–^ CM and other monocytes in PDDM patients. IM, intermediate monocytes; CM, classical monocytes; PD, Periodontitis; PDDM, Periodontitis and type II diabetes.

Subsequently, we performed cell-cell communication analysis and metabolic pathway activity analysis. Based on our previous results, LRRC25 was a marker gene of intermediate monocytes and NCF1 was a marker gene of classical monocytes. We further divided intermediate monocytes (IM) into two subgroups ( $LRRC25^{+}$ IM and $LRRC25^{-}$ IM), based on the expression of corresponding genes. Similarly, classical monocytes (CM) were divided into two subgroups ( $NCF1^{+}$ IM and $NCF1^{-}$ IM). In patients with periodontitis and patients with T2D and periodontitis, we observed that $LRRC25^{+}$ IM exhibited normal intercellular communications with other cell clusters, mainly through LGALS9 associated signaling pathways. However, $LRRC25^{-}$ IM did not exhibit any intercellular communications with other cell clusters (**Figures 10A, B, E, F**). In patients with periodontitis, we observed that $NCF1^{+}$ IM exhibited richer intercellular communication than $NCF1^{-}$ IM (**Figures 10C, D**). Similarly, there was significant difference in the intercellular communications between $NCF1^{+}$ IM and $NCF1^{-}$ IM in patients with T2D and periodontitis. $NCF1^{-}$ IM did not present any intercellular communications with other cell clusters at all (**Figures 10G, H**). The metabolic pathway activity analysis also indicated significant differences in metabolic pathway activity among different cell clusters. Overall, cell clusters expressing the two core genes exhibited heightened metabolic pathway activity (**Figures 10I–L**).

## 4 Discussion

To our knowledge, this is the first study to integrate various genetic approaches including single-cell analysis, MR analysis and colocalization analysis to delve into the association between T2D and periodontitis, and identify novel therapeutic targets. Single-cell analysis highlighted the importance of monocytes, with classical monocytes and intermediate monocytes identified as core cell subclusters. Subsequently, we obtained 221 marker genes as DEGs for MR analysis. 13 genes exhibited causal associations with T2D, and 11 genes demonstrated causal associations with periodontitis. Remarkably, among the 23 causal genes, LRRC25 presented causal associations with both diseases. To bolster the robustness of our findings, we conducted colocalization analysis, reverse MR analysis, additional validation and phenotype scanning. Finally, NCF1 and LRRC25 were identified as core therapeutic targets. To provide preliminary interpretations for the observed causal associations, we analyzed the expression of causal genes, intercellular communications among different cell clusters, and the metabolic pathway activity of these cell clusters. Significant differences of cell-cell communications and metabolic pathway activity were observed between monocytes expressing or not expressing the core genes, which might shed lights on the underlying mechanisms driving these causal associations.

Monocytes and their derived macrophages are vital components of the immune system, including classical monocytes, non-classical monocytes, and intermediate monocytes, which serve crucial roles in the body’s inflammatory response and vasculature surveillance (47). Existing studies indicate that monocytes and macrophages are highly engaged in the pathogenesis of T2D and periodontitis. Nagareddy et al. demonstrated that in obese mice, there was a notable elevation in monocyte levels, accompanied by the infiltration of their differentiated macrophages in adipose tissue. This infiltration contributed to the exacerbation of insulin resistance (IR) (48). Lira-Junior et al. revealed that S100A12 secreted by monocytes accumulated in inflammatory tissues and served as an indicator of the severity of periodontitis (49). As is well known, excessive monocytes can lead to the dysregulation of the body’s inflammatory responses, resulting in the release of large quantities of pro-inflammatory factors such as TNF- α , IL-1, and IL-6. On the one hand, this will promote the progression of T2D. On the other hand, it will also contribute to the occurrence of complications including periodontitis (44, 49). Therefore, delving into the crucial marker genes of monocytes and investigating their causal associations with T2D and periodontitis is of great significance for identifying novel therapeutic targets. Our study identified the core monocyte subclusters through single-cell analysis and selected important marker genes. MR analysis and colocalization analysis further investigated the causal relationships between genes and diseases, and we ultimately identified NCF1 and LRRC25 as core therapeutic targets.

Neutrophil cytosolic factor 1 (NCF1), a subunit of NADPH oxidase 2 (NOX2), possesses the ability to convert oxygen into superoxide anions and has been reported to be implicated in the pathogenesis of various diseases (50). For example, Geng et al.’s study indicated that NCF-H90, a lupus causal variant, might increase autoantibody production and contribute to kidney damage (51). In addition, several studies have established a strong correlation between NCF1 and renal fibrosis as well as rheumatoid arthritis (52, 53). In our study, NCF1 was identified as a risk causal gene for T2D, which exhibited significant colocalization with T2D. Currently, there are few studies reporting the role of NCF1 in T2D. Liu et al. demonstrated that reactive oxygen species (ROS) could increase the risk of type I diabetes by promoting the activation of autoreactive CD8+T cells. However, NCF1-mutate dendritic cells (DCs) exhibited diminished capacity to activate CD8+T cells (54). Based on these findings, we speculate that the mechanisms by which NCF1 increases the risk of T2D may also be related to CD8+T cells. Furthermore, we have observed that classical monocytes with high expression of NCF1 exhibited stronger intercellular communications compared to monocytes with low NCF1 expression (**Figure 10C**), which were primarily mediated through the LGALS9 signaling pathways (**Figure 10D**). These findings provide valuable insights into the causal relationship between NCF1 and T2D. In the future, it is possible that safe NCF1 inhibitors could be employed for the treatment of T2D.We anticipate that NCF1, a novel therapeutic target for T2D, will bring about greater well-beings for patients.

Leucine rich repeat containing 25 (LRRC25), a member of leucine rich repeat (LRR) containing protein family, is a key negative regulator in the signaling pathways of RIG-I-like receptors (RLRs) and type I interferon (IFN) (55). Currently, LRRC25 is recognized as a negative regulator within NF-κB signaling pathway. In addition, LRRC25 has been demonstrated to effectively inhibit inflammatory responses induced by lipopolysaccharide (LPS) and TNF- α (56, 57). In our study, LRRC25 was the only gene that exhibited causal association with both T2D and periodontitis, which had the potential to mitigate the risk of the two diseases. At present, there are few studies focusing on the role of LRRC25 in PD and T2D. Based on existing research, we speculate that the mechanisms by which LRRC25 reduces the risk of T2D and periodontitis may be intricately linked to the inhibition of inflammatory responses. Furthermore, a study focusing on acute myeloid leukemia (AML) has indicated that, in AML patients, there is a significant decrease in the proportion of monocytes, while LRRC25 exhibits high expression in primary bone marrow cells (granulocytes and monocytes) but low expression in lymphocytes. Additionally, they have demonstrated the crucial role of LRRC25 in inducing granulocyte differentiation (58). In our study, the proportion of monocytes in the PD group is decreased compared to the CT group, which was similar to that in AML patients. However, intriguingly, the PDDM group exhibited an increase in the proportion of monocytes, which necessitated further molecular biology experiments for elucidation. Looking ahead, as the results of basic experiments mature, we anticipate further clinical trials to validate the role of LRRC25 in monocytes in these two diseases. For patients with both T2D and periodontitis, we believe that LRRC25 will be a reliable therapeutic target, and more drugs targeting LRRC25 will be developed in the future.

Our study has several notable strengths. Firstly, this is the first study to integrate diverse genetic approaches to explore the intricate association between T2D and periodontitis, and we identified novel therapeutic targets for precise treatment of the two diseases. Secondly, compared to traditional clinical studies and bioinformatics analysis, MR analysis was employed in our study, which could effectively mitigate the impact of confounding factors and reverse causal bias. Thirdly, in MR analysis, the F-statistics of all IVs were greater than 10, indicating that weak instrument bias did not exist. At last, colocalization analysis, reverse MR analysis, additional validation and phenotype scanning were utilized in our study to enhance the robustness of our findings. Consequently, core therapeutic targets NCF1 and LRRC25 were identified.

However, it is crucial to acknowledge several limitations. Firstly, we did not conduct multiple testing. Our goal is to discover as many causal therapeutic targets related to T2D and periodontitis as possible, while multiple testing may exclude some meaningful indications. Secondly, the eQTLs and GWAS data are all from the European ancestry. Therefore, caution is needed when generating these results to other ethnic groups. Thirdly, Due to the lack of data on periodontitis, we did not verify the causal relationship between LRRC25 and periodontitis. In future investigations, once GWAS data from other races and additional periodontitis GWAS data are available, we will further validate our findings to fortify the robustness of our findings. At last, it is crucial to emphasize that although our study provides preliminary evidence of potential causal associations among 23 therapeutic targets, T2D and periodontitis, further experimental validation is still imperative to solidify these findings. For future research, cell line experiments and animal models could be used to further validate our findings.

## 5 Conclusion

In summary, this study preliminarily explored the association between T2D and periodontitis from a cellular and genetic perspective. Notably, classical monocytes and intermediate monocytes are regarded as core cell subclusters and may shed lights on the shared mechanisms in the pathogenesis of the two diseases. MR analysis identified 13 causal genes of T2D and 11 causal genes of periodontitis. Among them, NCF1 and LRRC25 were identified as core therapeutic targets. NCF1 presented colocalization with T2D and passed additional validation, and LRRC25 demonstrated causal association with both diseases. Furthermore, our analysis also suggests that monocytes expressing or not expressing the core genes exhibit different intercellular communications and metabolic pathway activity. Our findings provide novel insights into personalized treatment and targeted therapy. However, further biological experiments are still necessary for further validation.

## Data Availability

The original contributions presented in the study are included in the article/**Supplementary Material**. Further inquiries can be directed to the corresponding author.
